# Dr. John H. MacCallum

**Published:** 1918-04

**Authors:** 


**Dr. John H. MacCallum,** Pulte Medical College, 1862, died in Rochester, Feb., age 64.

